# Decoding temporal thermogenesis: coregulator selectivity and transcriptional control in brown and beige adipocytes

**DOI:** 10.1080/21623945.2024.2391511

**Published:** 2024-08-18

**Authors:** Yong Geun Jeon, Sun Won Kim, Jae Bum Kim

**Affiliations:** Center for Adipocyte Structure and Function, Institute of Molecular Biology and Genetics, School of Biological Sciences, Seoul National University, Seoul, South Korea

**Keywords:** Thermogenesis, brown adipocyte, beige adipocyte, co-regulators, RNF20

## Abstract

In mammals, brown adipose tissue (BAT) and beige adipocytes in white adipose tissue (WAT) play pivotal roles in maintaining body temperature and energy metabolism. In mice, BAT quickly stimulates thermogenesis by activating brown adipocytes upon cold exposure. In the presence of chronic cold stimuli, beige adipocytes are recruited in inguinal WAT to support heat generation. Accumulated evidence has shown that thermogenic execution of brown and beige adipocytes is regulated in a fat depot-specific manner. Recently, we have demonstrated that ubiquitin ligase ring finger protein 20 (RNF20) regulates brown and beige adipocyte thermogenesis through fat-depot-specific modulation. In BAT, RNF20 regulates transcription factor GA-binding protein alpha (GABPα), whereas in inguinal WAT, RNF20 potentiates transcriptional activity of peroxisome proliferator-activated receptor-gamma (PPARγ) through the degradation of nuclear corepressor 1 (NCoR1). This study proposes the molecular mechanisms by which co-regulator(s) selectively and temporally control transcription factors to coordinate adipose thermogenesis in a fat-depot-specific manner. In this Commentary, we provide molecular features of brown and beige adipocyte thermogenesis and discuss the underlying mechanisms of distinct thermogenic processes in two fat depots.

## Introduction

In mammals, adipose tissue plays key roles in maintaining energy homoeostasis and body temperature [1,2]. Adipose tissues are largely divided into white adipose tissue (WAT) storing extra energy in the form of triglycerides and brown adipose tissue (BAT) producing heat. Also, in mice, brown-like thermogenic adipocytes called beige (or brite) adipocytes are recruited in multiple WATs such as axillary, suprascapular, periovarian, and inguinal WAT (iWAT) upon several metabolic stimuli such as prolonged cold exposure [3–6]. Key features of thermogenic brown and beige adipocytes are abundant mitochondria, multilocular lipid droplets (LDs), and high expression of uncoupling protein 1 (UCP1), which primarily mediates nonshivering thermogenesis [7–10].

**Figure 1.**
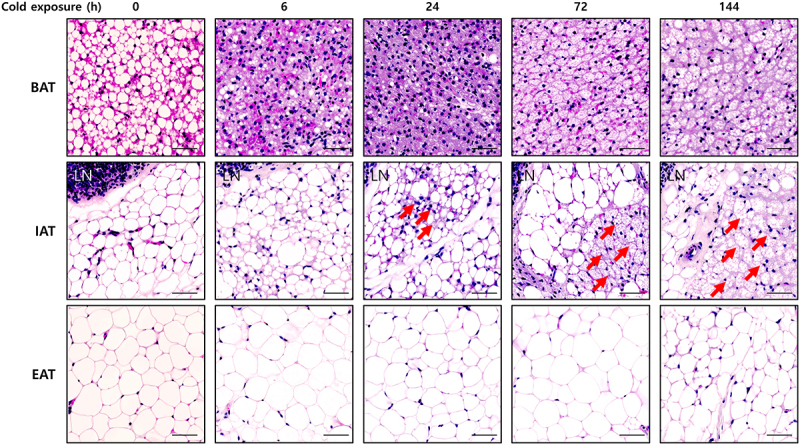
Upon cold exposure, BAT, iWAT, and eWAT respond differently. Representative H&E staining images of BAT, iWAT, and eWAT. Upon acute cold stimuli (6 h, 6°C), the size of lipid droplets (LDs) in brown adipocytes is rapidly reduced. Upon prolonged cold stimuli (72 h and 144 h), the shape of brown adipocytes is different from that in the absence of cold stimuli with highly multilocular lipid droplets. Unlike BAT, multilocular beige adipocytes are not present in iWAT upon acute cold stimuli (6 h). At 24 h cold stimuli, multilocular beige adipocytes (red arrow) are recruited in iWAT. In addition, 72 h and 144 h cold stimuli stimulate beige adipocyte recruitment. In contrast, multilocular beige adipocytes are not observed in eWAT. C57BL/6 male mice are housed at room temperature (22°C) and then exposed to cold stimuli. Until 6 h cold stimuli, mice were fasted. Scale bar: 50 µm. LN: lymph node.

In rodents, brown adipocytes are constitutively present and rapidly activated to generate heat upon acute cold stimuli, along with skeletal muscle-derived shivering thermogenesis [10]. During prolonged cold stimuli, the levels of mitochondrial contents and lipid droplets in brown adipocytes are increased [10] (Figure 1), as muscle shivering thermogenesis is decreased. Also, the number of brown adipocytes is elevated by proliferation and differentiation of brown adipocyte precursors, especially in the dorsal region of BAT [11]. Human infants have brown adipocytes in the interscapular and perirenal regions [12] and adult humans have thermogenic adipocytes in the cervical, supraclavicular, and axillary regions, which are activated by cold stimuli [13].

WATs also control body temperature through their unique properties during cold stimuli. In mice, UCP1-positive multilocular beige adipocytes are recruited to iWAT in the presence of cold stimuli for more than 1 day (Figure 1). Beige adipocytes can be recruited by *de novo* beige adipogenesis from beige adipocyte precursors and transformation of UCP1-negative adipocytes (or activation of dormant beige adipocytes) into beige adipocytes [14]. On the other hand, in several rodent visceral WATs such as mesenteric, retroperitoneal, and epididymal WAT (eWAT), recruitment of beige adipocytes is repressed upon cold stimuli (Figure 1). Instead, adipocytes within these non-thermogenic fat depots appear to supply lipid metabolites through lipolysis [15]. Together, these findings suggest that each fat depot plays a different role in maintaining the body temperature in response to cold stimuli through distinct molecular mechanisms [16].

In this Commentary, we focus on the mechanisms underlying the unique thermogenic role of each fat depot. To this end, we will describe the developmental origins of brown and beige adipocytes and the distinct mechanisms that could control thermogenesis in brown and beige adipocytes. Furthermore, we will discuss recent findings that the selectivity of coregulators would be important for fat-depot selective thermogenesis.

## Development of brown and beige adipocytes

During the developmental process, BAT is the firstly developed adipose tissue and originated from dorsomedial dermomyotomes expressing *Myf5*, *En1*, and *Pax7,* as well as a subset of central dermomyotomes [17–20]. In mice, peroxisome proliferator-activated receptor-gamma (PPARγ)-expressing brown adipocyte progenitors are formed at embryonic day 14.5 (E14.5) [21,22], and perilipin 1 (PLIN1)-expressing brown adipocytes are present at E15.5 [21]. Accumulating evidence has suggested that PPARγ, CCAAT/enhancer-binding protein-beta (C/EBPβ), and PPARγ co-activator-1 alpha (PGC-1α) are core transcriptional regulators for brown adipocyte development [23]. In addition, the PR domain containing 16 (PRDM16) promotes brown adipocyte differentiation by forming a transcriptional complex with PPARg and C/EBPb to stimulate the expression of brown adipocyte-specific genes and represses myogenesis by suppressing myogenic differentiation 1 transcription factor [20,23]. Among numerous factors, PPARγ plays a crucial role in maintaining BAT activity and identity in the developmental stage, as well as in adult mice [24,25]. Furthermore, recent studies have proposed that the transcription factor GATA-binding factor 6 marks brown adipocyte progenitor cells and induces PPARγ expression for brown adipocyte development [21,22]. In addition, several transcriptional and epigenetic regulators such as ZFP423, ZFP516, EBF2, IRF4, and TLE3 also regulate brown adipogenesis (please refer to review [23])

iWAT is developed from splanchnic posterior lateral plate mesoderm at E14.5 from *Prrx1* and/or *Hoxb6*-expressing progenitor cells [26–29]. Lineage-tracing studies have revealed that beige adipocytes are differentiated from a subpopulation of *Pdgfra*-positive and *Myf5*-negative adipocyte progenitor cells in iWAT [30,31]. Recently, single-cell RNA sequencing (scRNA-seq) analyses have shown that beige precursors are differentiated from CD81^+^ adipocyte progenitors or bone marrow stromal cell antigen 2 (BST2)^high^ adipocyte progenitors [32,33]. Intriguingly, biogenesis of beige adipocyte progenitors in iWAT appears to be promoted by secreted molecules of inguinal lymph node [33,34], implying that immune-progenitor crosstalk might be important for beige adipocyte development. In addition to *de novo* beige adipogenesis, beige adipocytes can be derived from the transformation of white adipocytes [14]. The proportion of *de novo* beige adipogenesis and transformation of white adipocytes is affected by the types of cold stimuli (e.g. cold or β-adrenergic agonist), housing temperature, and experience of cold cycle [14], which would affect epigenetic memory in white adipocytes [35].

## Molecular mechanisms of brown adipocyte activation upon acute cold stimuli

**Figure 2.**
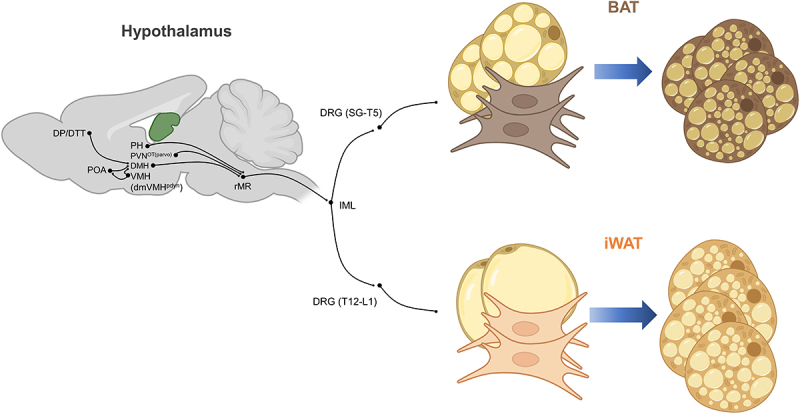
Neural pathways contributing to thermogenesis. Diverse hypothalamic subregions such as preoptic area of the hypothalamus (POA), posterior hypothalamus (PH), parvocellular oxytocin neurons of the PVN (PVN^OT(parvo)^), dorsomedial hypothalamus (DMH), pdyn-expressing dorsal medial region of the ventromedial hypothalamus (dmVMH^Pdyn^), and the dorsal peduncular/dorsal tenia tecta (DP/DTT) region of the prefrontal cortex activate thermogenesis. Sympathetic premotor neurons in the rostral medullary raphe (rMR) control thermogenesis via projections to the intermediolateral nucleus (IML) spinal cord, which activate dorsal root ganglia (DRG), thereby activating BAT and iWAT. SG: stellate ganglia, T: thoracic, L: lumbar. The figure was created with BioRender.com.

Brown and beige adipocytes function as the thermoregulatory defence system against hypothermia through sequential activation. Upon acute cold stimuli (less than 1 day), BAT rapidly stimulates heat generation by activating pre-existing brown adipocytes [10,36], thereby playing the first line of defence against hypothermia. In response to cold, thermosensitive TRPM8 in the skin is activated, initiating a neural response that is relayed to the hypothalamus [37]. The preoptic area of the hypothalamus integrates the cold stimuli and activates the dorsomedial hypothalamus, which then stimulates rostral medullary raphe (Figure 2) [38]. Additionally, the posterior hypothalamus, paraventricular nucleus of the hypothalamus, and ventral medial hypothalamus are linked to thermoregulatory neuronal circuits [38,39]. This neuronal cascade triggers the sympathetic nervous system (SNS), leading to the release of norepinephrine (NE) from sympathetic nerve terminals innervating BAT. Subsequently, the released NE binds to β1, β2, and β3-adrenergic receptors (ARs) on brown adipocytes [40–44]. At the lower level of NE concentration, β1AR is first to be activated [45], and at higher NE concentration, more abundant rodent β3AR [45] and human β2AR [46] become activated to initiate intracellular signalling cascades for thermogenesis [10,40,47,48].

**Figure 3.**
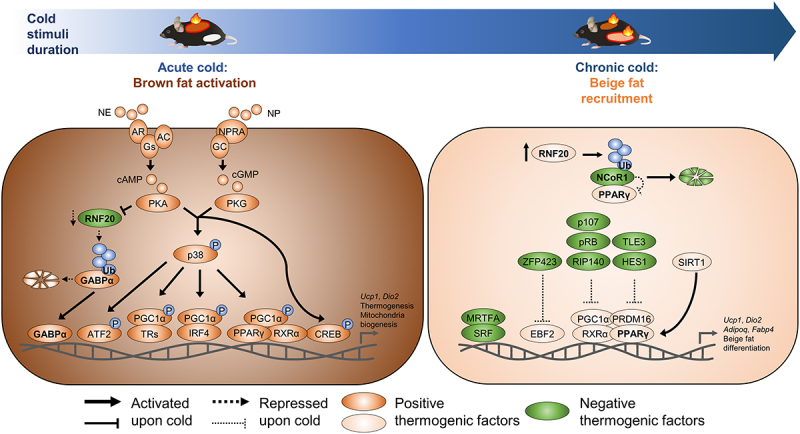
Molecular mechanisms of distinct brown and beige fat thermogenesis during cold stimuli. Upon cold stimuli, mammals maintain their body temperature by enhancing thermogenesis in several fat depots. Sympathetic neurons release norepinephrine (NE), which binds to β-adrenoreceptors (AR). This binding activates adenylyl cyclase (AC), elevating cyclic AMP (cAMP) levels and increasing PKA activity. Additionally, natriuretic peptides (NP) bind to natriuretic peptide receptor a (NPRA), which activates guanylyl cyclase (GC) to increase cyclic GMP (cGMP) concentrations, leading to PKG activation. Both activated PKA and PKG phosphorylates CREB and p38 MAPK. Further, p38 MAPK phosphorylates and activates ATF2 and PGC-1α, promoting the expression of thermogenic genes. PGC-1α interacts with thyroid receptors (TRs), PPARγ/RXRα, and IRF4 to stimulate the expression of thermogenic genes. Moreover, acute cold exposure rapidly downregulates RNF20 via PKA signalling, leading to GABPα accumulation and activation. In turn, GABPα contributes to potentiating brown fat thermogenesis by stimulating the expression of thermogenic and mitochondrial genes. In iWAT, prolonged cold exposure gradually upregulates RNF20, which activates PPARγ by degrading NCoR1, promoting beige fat recruitment. PPARγ binds with EBF2, PGC-1α, and PRDM16, which stimulates the expression of adipogenic and thermogenic genes in beige fat. Key transcriptional co-regulators controlling genetic programming for beige fat recruitment are also depicted. Gs: G protein subunit.

The activation of β3ARs leads to stimulation of adenylate cyclase, increasing intracellular cyclic AMP (cAMP) levels and activating protein kinase A (PKA) (Figure 3) [10,49]. Then, PKA activates the p38 mitogen-activated protein kinase (MAPK) pathway, leading to the phosphorylation of activating transcription factor 2 (ATF2), which translocates to the nucleus and promotes transcription of UCP1 [50]. Moreover, PKA phosphorylates cAMP response element-binding protein (CREB), further potentiating UCP1 expression [50]. In addition, the p38 phosphorylation of ATF2 drives the expression of PGC-1α [50], which interacts with PPARγ and oestrogen-related receptor alpha (ERRα) to augment UCP1 gene expression and mitochondrial biogenesis [51]. Furthermore, PGC-1α interacts with thyroid hormone receptors and interferon-related factor 4 to induce thermogenic gene expression [10,52].

In parallel, cardiac natriuretic peptides contribute to thermogenic activation of BAT. Upon acute cold stimuli, circulating levels of cardiac natriuretic peptides are elevated about 10-fold [53], which facilitates thermogenic and mitochondrial gene expression in BAT. Mechanistically, cardiac natriuretic peptides activate cyclic GMP-dependent protein kinase (PKG), which phosphorylates p38 MAPK and ATF2 to promote thermogenic program in BAT [53].

As protein phosphorylation is often temporal and transient, other relatively rapid and stable mechanisms are required to potentiate thermogenic gene expression and mitochondrial biogenesis upon acute cold stimuli. It has been reported that the proteasomal activity in BAT is altered upon cold stimuli [54], implying that protein stability control would be one of the molecular mechanisms to maintain thermogenic activity. For instance, several E3 ubiquitin ligases such as MKRN, RNF34, and synoviolin are involved in thermogenic gene expression through protein regulation of AMPK [55], PGC-1α [56], and PGC-1β [57], respectively, in brown adipocytes and/or BAT. Recently, we have identified that another E3 ubiquitin ligase ring finger protein 20 (RNF20) contributes to acute thermogenic activation in BAT [58]. Upon acute cold stimuli or β-adrenergic stimuli, RNF20 in brown adipocytes is rapidly downregulated, which promotes BAT thermogenic gene expression in BAT. Furthermore, we have identified that RNF20 in BAT promotes the proteasomal degradation of GA-binding protein alpha (GABPα) (Figure 3). Using BAT-specific overexpression and knockdown of GABPα, we have elucidated that GABPα plays a key role in the regulation of thermogenic and mitochondrial biogenesis genes in BAT. These findings suggest that RNF20-GABPα axis ensures rapid and efficient BAT thermogenesis during acute cold stimuli.

## Molecular mechanisms of beige adipogenesis upon prolonged cold stimuli

In animals, WAT, especially subcutaneous WAT, also contributes to maintaining body temperature. Several terrestrial and marine animals living in cold environments such as polar bears, tuna, and blue marlin prevent hypothermia with a large amount of subcutaneous WAT as a heat insulator [59,60]. In addition, subcutaneous WAT, especially iWAT in rodents, is able to produce heat by recruiting beige adipocytes upon prolonged cold stimuli [41,61–63].

The duration of cold stimuli is an important factor in the recruitment of beige adipocytes [26,58,64]. In mice housed at room temperature (20–23°C) or thermoneutral conditions (28–30°C), a few multilocular beige adipocytes are present in iWAT, which are abundantly located around lymph nodes [14,33]. Upon acute cold stimuli (less than 1 day), secreted NE from sympathetic nerve terminals innervating iWAT promotes lipolysis in white adipocytes to provide energy sources [58]. After 1 day of cold exposure, beige adipocytes begin to be recruited in iWAT, and cold stimuli for more than 3 days significantly promote beige adipocyte recruitment [34,58]. Similarly, β3-adrenergic agonist CL316,243 administration for more than 3 days potentiates beige adipocyte recruitment in iWAT [65].

Accumulating evidence suggests that PPARγ plays crucial roles in beige adipocyte recruitment for both *de novo* beige adipogenesis and transformation of white adipocytes [66]. PPARγ forms a heterodimer with retinoid X receptor alpha (RXRα) and directly binds to the UCP1 promoter to enhance UCP1 expression as well as other adipogenic genes (Figure 3) [67]. In addition, the interaction between PPARγ and PGC-1α and/or PRDM16 facilitates mitochondrial biogenesis and function, ensuring a robust thermogenic gene expression [23]. In line with these, selective PPARγ deletion in CD81-expressing beige adipocyte progenitors impairs thermogenic gene expression in iWAT upon prolonged cold stimuli [21]. Furthermore, it has been well established that treatment with PPARγ agonists such as rosiglitazone in differentiated adipocytes and mice stimulates thermogenic gene expression [23,68,69]. Mechanistically, it has been reported that rosiglitazone treatment stabilizes PRDM16 protein [68] and triggers the sirtuin 1 (SIRT1)-dependent deacetylation of PPARγ, which enhances the interaction between PRDM16 and PPARγ to potentiate the expression of thermogenic genes [70].

Despite these findings, temporal regulatory mechanisms that facilitate PPARγ activity in response to prolonged cold stimuli are largely unknown. Our recent findings have suggested that RNF20 in iWAT is gradually upregulated upon chronic cold stimuli, which leads to activation of PPARγ by decreasing nuclear corepressor 1 (NCoR1) for *de novo* beige adipogenesis (Figure 3) [58]. We and others have previously elucidated that RNF20 stimulates NCoR1 degradation in epididymal WAT and 3T3-L1 white adipocytes [71,72]. Consistent with these, in iWAT, RNF20 promotes NCoR1 poly-ubiquitination and degradation. Thus, upon prolonged cold stimuli, upregulated RNF20 decreases the level of NCoR1 protein in iWAT, leading to upregulation of PPARγ target genes for beige adipogenesis [58]. Furthermore, *in vitro* cell culture models and *in vivo* adipocyte-chasing mice experiments have shown that RNF20 promotes *de novo* beige adipogenesis in iWAT [58]. Given that RNF20-NCoR1 axis regulates PPARγ activity in iWAT, it is plausible to speculate that RNF20 upregulation might also stimulate the transformation of white adipocytes into beige adipocytes. Together, these findings indicate that RNF20 would act as a physiological regulator for beige adipocyte recruitment upon prolonged cold stimuli.

## Distinct molecular features of brown and beige adipocytes

Brown adipocytes and beige adipocytes exhibit overlapping but different transcriptomic features. Although *Ucp1* and *Pgc1a* are highly expressed in both beige and brown adipocytes, mouse beige adipocytes selectively express several markers such as *Tmem26* and *Tbx1* [35,73,74], whereas brown adipocytes specifically express selective markers including *Zic1 and Slc29a1* [35,73]. Nonetheless, as many studies have been performed using *in vitro* cell lines, further investigation using single-cell level and spatial RNA sequencing analyses of BAT and iWAT [75,76] may shed light on the common and distinct molecular profiles of brown and beige adipocytes.

Several transcriptional and epigenetic regulators play distinct roles in brown and beige adipocytes. For example, transcriptional coactivator myocardin-related transcription factor A (MRTFA) suppresses beige adipocyte recruitment but does not affect BAT thermogenic gene program through inhibition of commitment to the beige adipogenic lineage of mesenchymal stem cells [77]. Also, transcriptional repressors including HES1, Rb, and p107 suppress PGC-1α transcription [78,79], and transcriptional modulators such as TLE3 and HES1 regulate PRDM16 activation [80] or transcription [78] in iWAT. In addition, the nuclear corepressor receptor-interacting protein 140 (RIP140) suppresses PGC-1α, and SIRT1 induces PPARγ activity, respectively, in iWAT but not in BAT [70,81,82]. Moreover, our recent data have revealed that RNF20 suppresses thermogenic gene program in BAT, whereas it potentiates beige adipogenesis in iWAT [58]. These tissue-specific roles of several transcriptional regulators are likely to be crucial for the distinct roles of brown and beige adipocytes upon cold duration.

Although the underlying mechanisms for the distinct roles of several thermogenic regulators have not been well understood, it is noteworthy that MRTFA, HES1, RIP140, SIRT1, and RNF20 are co-regulators modulating the activity of several transcription factors. It is feasible to speculate that target transcription factors of these coregulators might be different in BAT and iWAT. For instance, RNF20 primarily regulates GABPα stability and activity in BAT, whereas it controls NCoR1 stability to activate PPARγ in iWAT. Thus, it is likely that different fat-depot-specific regulators might have their own target transcription factor(s) in a tissue-specific manner. Moreover, as the level of RNF20 protein is differently regulated in BAT and iWAT upon cold stimuli, it seems that transcriptional and post-translational modifications of these regulators may vary in fat depots upon cold exposure [83]. Therefore, it will be important to elucidate the underlying mechanisms that control fat depot-specific signalling pathways in future studies.

## Conclusions and perspectives

Proper responses to external stimuli according to their intensity and duration are essential for the survival of most organisms. For instance, when pathogens invade, the acute-phase responses of innate immunity protect the organism from infection. Then, the pathogen experience is memorized by adaptive immunity, which plays crucial roles in efficiently combating future pathogen invasions [84]. Similarly, with acute cold exposure, the rapid activation of pre-existing brown adipocytes is imperative for maintaining body temperature, and upon chronic cold stimuli, the recruitment of beige adipocytes facilitates adaptive thermogenesis to sustain thermogenic capacity. Therefore, it is reasonable to assume that there should be fat-depot-specific regulatory mechanisms for the effective and sufficient activation of brown adipocytes and the recruitment of beige adipocytes depending on the intensity and duration of external cold stimuli.

Our recent data propose that RNF20 modulates brown and beige adipocyte thermogenesis through different fat depot-specific regulatory processes, implying the unique roles and distinct regulatory mechanisms of brown and beige adipocytes. Nonetheless, the tissue-specific regulation of RNF20 and the molecular mechanisms driving tissue-specific substrate preferences warrant further investigation. Exploring these questions will broaden our understanding of the specific roles of fat depots and the molecular mechanisms that regulate RNF20, which will expand our knowledge of the functions and regulatory mechanisms of thermogenic adipocytes.

## Data Availability

The analysed data used to support the findings of this study are included within the article. The figures are available on Figshare at https://doi.org/10.6084/m9.figshare.26401285.v2, https://doi.org/10.6084/m9.figshare.26401288.v2, and https://doi.org/10.6084/m9.figshare.26401291.v2 [85–87].
